# Draft genome sequence of the coccolithovirus EhV-84

**DOI:** 10.4056/sigs.1884581

**Published:** 2011-09-23

**Authors:** Jozef I. Nissimov, Charlotte A. Worthy, Paul Rooks, Johnathan A. Napier, Susan A. Kimmance, Matthew R Henn, Hiroyuki Ogata, Michael J. Allen

**Affiliations:** 1 Plymouth Marine Laboratory, Prospect Place, The Hoe, Plymouth, PL1 3DH, UK; 2 Department of Biological Chemistry, Rothamsted Research, Harpenden, Herts AL5; 2JQ; 3 The Broad Institute of MIT and Harvard, Cambridge, Massachusetts, United States; of America; 4 Structural and Genomic Information Laboratory, CNRS-UPR2589, Mediterranean Institute of Microbiology (IFR-88), Aix-Marseille University, 163 avenue de Luminy Case 934, FR-13288 Marseille, France

**Keywords:** coccolithovirus, marine, phycodnavirus, algae, virus

## Abstract

The *Coccolithoviridae* is a recently discovered group of viruses that infect the marine coccolithophorid *Emiliania huxleyi*. Emiliania huxleyi virus 84 (EhV-84) has a 160 -180 nm diameter icosahedral structure and a genome of approximately 400 kbp. Here we describe the structural and genomic features of this virus, together with a near complete draft genome sequence (~99%) and its annotation. This is the fourth genome sequence of a member of the coccolithovirus family.

## Introduction

Coccolithoviruses infect the cosmopolitan marine microalgae, *Emiliania huxleyi* [1]. These algae are capable of forming vast blooms which can be seen from space and can cover up to 100, 000 km^2^ occurring in the top 50-100 m of the water column, with a cellular density of more than a million cells per liter of seawater [2]. *E. huxleyi* has become a species crucial to the study of global biogeochemical cycling [3-5]. The elegant calcium carbonate scales (known as coccoliths) which it produces intracellularly and the scale of its blooms have made *E. huxleyi* an essential model organism for marine primary productivity and global carbon cycling [6]. Coccolithoviruses have been shown to be a major cause of coccolithophore bloom termination and their pivotal role in global biogeochemical cycling has gained increasing attention. Coccolithovirus abundances typically reach 10^7^ per ml in natural seawater under bloom conditions and 10^8^ -10^9^ per ml under laboratory culture. The model coccolithovirus strain EhV-86 (AJ890364), and two other similar but genetically distinct strains, EhV-84 and EhV-88 were isolated in 1999 from a coccolithophore bloom in the English Channel. EhV-86 was sequenced in its entirety in 2005 to reveal a genome of 407,339 bp. Two further strains, EhV-163 and EhV-99B1 were isolated in 2000 and 1999 respectively from a Norwegian fjord and have had their partial genomes also sequenced [7,8]. All coccolithoviruses known to date have been isolated from the English Channel and a Norwegian fjord. Here we present a summary classification and a set of features for coccolithovirus strain EhV-84, the second English Channel coccolithovirus sequenced, together with the description of the sequencing and annotation of its genome.

## Classification and features

**Figure 1:**
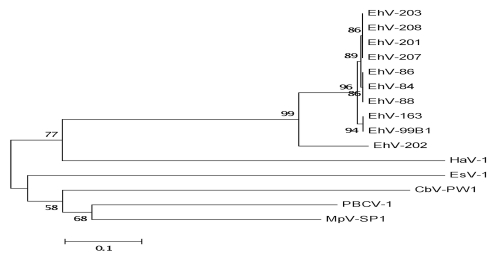
Multiple Sequence Alignment of the DNA pol (DNA polymerase) gene of ten coccolithoviruses (EhVs) and five other algal viruses. The evolutionary history was inferred using the Neighbor-Joining method [9]. The bootstrap consensus tree inferred from 1000 replicates is taken to represent the evolutionary history of the taxa analyzed [10]. The percentage of replicate trees in which the associated taxa clustered together in the bootstrap test (1000 replicates) are shown next to the branches when greater than 50% [10]. The evolutionary distances were computed using the Maximum Composite Likelihood method [11] and are in the units of the number of base substitutions per site. All positions containing gaps and missing data were eliminated from the dataset (Complete deletion option). Phylogenetic analyses were conducted in MEGA4 [12].

**Figure 2:**
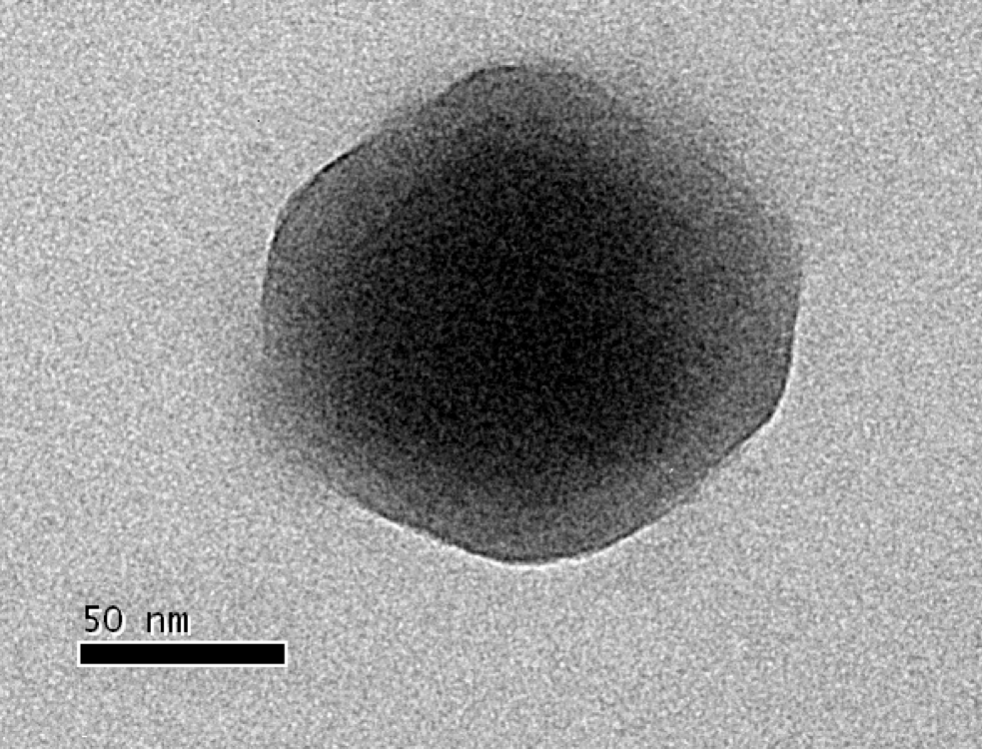
Transmission electron micrograph of an EhV-84 virion.

All coccolithoviruses to date have been isolated from *E. huxleyi* algal blooms in temperate and sub temperate oceanic waters. Maximum likelihood phylogenetic analysis of available DNA polymerase gene sequences (DNA pol), one of the viral kingdom’s phylogenetic markers (equivalent to 16S rDNA sequences in bacteria) indicates that the closest related viral strain to EhV-84 is EhV-86 and EhV-88 (Figure 1). Both of these strains were isolated from the English Channel in the same year as EhV-84 [13]. The English Channel EhVs that were isolated in 1999 (EhV-84, EhV-86 and EhV-88) are more similar to other strains from the English Channel such as EhV-201, EhV-203, EhV-207 and EhV-208 isolated two years later in 2001, than strains such as EhV-163 and EhV-99B1 that are from a different geographical location; i.e. a Norwegian fjord. Interestingly EhV-202 seems to be the most different of all strains sequenced to date and this is also evident from full genome sequencing (data not published). Other algal viruses such as Paramecium bursaria Chlorella virus (PBCV-1), Micromonas pusilla virus SP1 (MpV-SP1), Chrysochromulina brevifilum virus PW1 (CbV-PW1), Ectocarpus siliculosus virus 1 (EsV-1), Heterosigma akashiwo virus 01 (HaV-01) are included here as an additional reference and they cluster outside the EhVs genus. The EhV-84 virion structure has icosahedral morphology, a diameter of 160 -180 nm (Figure 2), and is similar to other coccolithoviruses (and phycodnaviruses in general) [14]. Isolation and general phylogenetic characteristics are outlined in Table 1.

**Table 1:** Classification and general features of Emiliania huxleyi virus 84 according to the MIGS recommendations [15].

**MIGS ID**	**Property**	**Term**	**Evidence code**
	Current classification	Domain: Viruses, dsDNA viruses, no RNA stage Class: NCLDNA **(**Nucleo**-**Cytoplasmic Large DNA) Family: *Phycodnaviridae* Genus: *Coccolithovirus* Species: Emiliania huxleyi virus 84	TAS [16] TAS [16] TAS [16] TAS [16] TAS [16]
	Virion shape	Icosahedral	IDA
MIGS-6	Habitat	Oceanic, Coastal	TAS [16]
MIGS-15	Biotic relationship	Obligate intracellular parasite of *Emiliania huxleyi*	TAS [16]
MIGS-14	Pathogenicity	Lytic virus of *Emiliania huxleyi*	TAS [16]
MIGS-4	Geographic location	English Channel, UK	TAS [16]
MIGS-5	Sample collection time	July 26, 1999	TAS [16]
MIGS-4.1 MIGS-4.2	Latitude Longitude	50.15 N 4.13 E	TAS [16]
MIGS-4.3	Depth	15 m	TAS [16]

Evidence codes - IDA: Inferred from Direct Assay; TAS: Traceable Author Statement (i.e., a direct report exists in the literature); NAS: Non-traceable Author Statement (i.e., not directly observed for the living, isolated sample, but based on a generally accepted property for the species, or anecdotal evidence). These evidence codes are from the Gene Ontology project [17].

## Genome sequencing and annotation

### Genome project history

The Marine Microbiology Initiative (MMI) of the Gordon & Betty Moore Foundation aims to generate new knowledge about the composition, function, and ecological role of the microbial communities that serve as the basis of the  food webs of the oceans and that facilitate the flow of nitrogen, carbon, and energy in the ocean. In an effort to understand the ecology and evolution of marine phage and viruses and to explore the diversity and ecological roles of entire phage/viral communities through metagenomics, the Broad Institute collaborated with MMI and researchers whose sequencing nominations were chosen by the Marine Phage, Virus, and Virome Selection Committee to generate genomic sequence and annotation of ecologically important phage. EhV-84 was nominated for sequencing on the basis of its global importance in the demise of *E. huxleyi* blooms [13], the horizontal gene transfer events observed in other coccolithovirus genomes [18], the metabolic potential displayed by its large genome size and its possible manipulation of signaling pathways such as programmed cell death in its host organism [8,19].

The genome project is deposited in the The Integrated Microbial Genomes (IMG) system and the complete genome sequence and annotation are available in GenBank (JF974290). Genome sequencing, finishing and annotation were performed by the Broad Institute. A summary of the project information is shown in Table 2.

**Table 2:** Genome sequencing project information

**MIGS ID**	**Property**	**Term**
MIGS-31	Finishing quality	Finished (>99%)
	Number of contigs	9
	Average contig size	43,980
	Largest contig size	97,445
	Assembly size (using large contigs)	395,820
	Assembly coverage ("peak Depth")	36.16
	Total number of reads used	28,526
MIGS-29	Sequencing platforms	454
MIGS-30	Assemblers	Newbler Version 2.3 PostRelease-11.19.2009
MIGS-32	Gene calling method	Broad Institute Automated Phage Annotation Protocol [20]
	GenBank ID	JF974290
	GOLD ID	N/A
	Project relevance	Gordon & Betty Moore Foundation's Marine Microbiology Initiative. Emiliania huxleyi virus 84- G3248.

### Growth conditions and DNA isolation

*Emiliania huxleyi* strain CCMP 2090 was grown in 1 liter cultures (f/2 nutrient media) in the laboratory under a light/dark cycle of 16/8 respectively, at a temperature of 16°C. Once the cultures were at mid exponential growth (i.e. 4 × 10^6^ ml^-1^), they were infected with an EhV-84 lysate at an MOI ratio of 1:1. Infection, host death and viral production were confirmed by flow cytometry. Fresh virus lysate was filtered through a 0.2 µm pore 47 mm diameter Durapore filter (Millipore). Viruses were concentrated by PEG precipitation, subjected to a CsCl gradient and the DNA extracted [8,21].

### Genome sequencing and assembly

The genome of strain EhV-84 was sequenced using the 454 FLX pyrosequencing platform (Roche/454, Branford, CT, USA). Library construction, and sequencing were performed as previously described [20]. General protocols for library construction can be found at [22]. *De novo* genome assembly of resulting reads was performed using the Newbler v2.3 assembly software package as previously described [20]. Assembly metrics are as described in Table 3.

**Table 3:** *De novo* assembly metrics for EhV-84

**No. of Reads Assembled**	**No. of Contigs**	**Largest Contig (bp)**	Total Contig Length (bp)*	**Average Contig Sequence Coverage**	**Percent of Bases Q40**
28526	9	97445	395820	36.9 ± 3.6	94.9

*Total contig length does not include bp for gaps of unknown size

### Genome annotation

Genes were identified using the Broad Institute Automated Phage Annotation Protocol as previously described [20]. In short, evidence based and *ab initio* gene prediction algorithms where used to identify putative genes followed by construction of a consensus gene model using a rules-based evidence approach. Gene models where manually checked for errors such as in-frame stops, very short proteins, splits, and merges. Additional gene prediction analysis and functional annotation was performed within the Integrated Microbial Genomes – Expert Review platform [23].

## Genome properties

**Figure 3:**
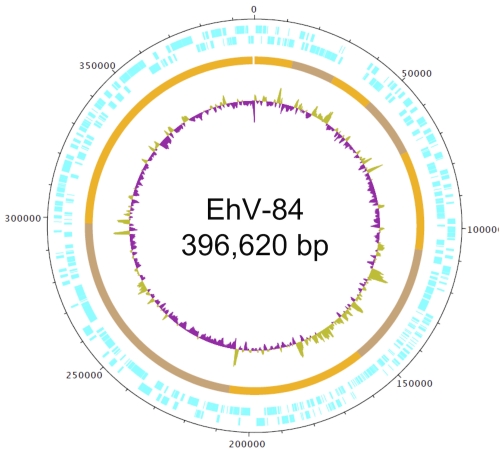
Graphical circular map of the 396,620 bp EhV-84 genome. The outside scale is numbered clockwise in bp. Circles 1 and 2 (from outside in) denotes CDSs (forward and reverse strands, respectively). Circle 3 represents the nine contigs of the genome that were used to construct the draft genome using the EhV-86 genome as the reference, and circle 4 is the G+C content.

General features of the EhV-84 genome sequence (Table 4) include a nucleotide composition of 40.17% G+C (Figure 3), a total of 482 predicted protein coding genes and four tRNA genes (encoding amino acids Arg, Asn, Gln and Ile). Of the 482 CDSs, 85 (17.49%) have been annotated with functional product predictions (Table 4) and the genes have been categorized into COGs functional groups (Table 5).

**Table 4:** Genome statistics of EhV-84

Attribute	Value	% of total^a^
Size (bp)	396,620	100.00%
G+C content (bp)	158,983	40.17%
Coding region (bp)	334,463	84.33%
Total genes^b^	486	100.00%
RNA genes	4	0.82%
Protein-coding genes	482	99.18%
Protein coding genes with function prediction	85	17.49%
Genes in paralog clusters	15	3.09%
Genes with signal peptides	142	29.22%

**^a^** The total is based on either the size of the genome in base pairs or the total number of protein coding genes in the annotated genome, where applicable.

**^b^** Includes 18 pseudogenes.

**Table 5:** Number of genes associated with COG functional categories.

Code	value	%age	Description
G	1	1.41	Carbohydrate transport and metabolism
D	3	4.23	Cell cycle control, cell division, chromosome partitioning
M	2	2.82	Cell wall/membrane/envelope biogenesis
H	2	2.82	Coenzyme transport and metabolism
S	6	8.45	Function unknown
R	7	9.86	General function prediction only
P	1	1.41	Inorganic ion transport and metabolism
U	2	2.82	Intracellular trafficking, secretion, and vesicular transport
I	5	7.04	Lipid transport and metabolism
F	6	8.45	Nucleotide transport and metabolism
O	8	11.27	Posttranslational modification, prot. turnover, chaperones
L	11	15.49	Replication, recombination and repair
A	4	5.63	RNA processing and modification
T	1	1.41	Signal transduction mechanisms
K	12	16.90	Transcription

## Insights from the genome sequence

### Comparative genomics

EhV-84 is now the fourth coccolithovirus strain to have its genome determined. EhV-84 displays a near identical G+C content to EhV-86; i.e. 40.17% and 40.18% respectively. EhV-84 is predicted to encode 482 coding sequences (including 18 pseudogenes) and four tRNA genes (Arg, Asn, Gln and Ile), whereas EhV-86 has 472 CDSs and five tRNAs (Arg, Asn, Gln, Ile and Leu). Two of the EhV-84 tRNAs are identical in length and sequence to tRNAs in EhV-86 (Gln, 72 bases; Asn, 74 bases), one is 98% similar (Arg, 72 bases in EhV-84; 73 bases in EhV-86). However, the Ile tRNA of EhV-84 varies dramatically, containing a 26 base intron insertion (99 bases in EhV-84; 73 bases in EhV-86). EhV-86 has an extra Leu (103 bases) that is absent from the genome of EhV-84.

There are 224 CDSs in EhV-84 which share 100% sequence identity (TBLASTN) with homologues in EhV-86. A further 198 CDSs have non-identical homologues in EhV-86, with similarities greater than 10%  (settings in IMG/ER: TBLASTN, Max e-value 1e-5, min. percent identity 10, algorithm by present/absent homologs, min. taxon percent with homologs 100, min. taxon percent without homologs 100). Of the CDSs shared between EhV-84 and EhV-86, 69 have an assigned function in EhV-86 that also corresponds to sequences in the Conserved Domain Database (Table 6). More than half (38/69) are identical in both strains. In addition, there are a further 60 annotated CDSs in EhV-84 which have no homologues in EhV-86, two of which have homologues in EhV-99B1 (ENVG00303 and ENVG00419, encoding a hypothetical protein and zinc finger protein, respectively). Three of the unique EhV-84 CDSs show similarity to sequences in the Conserved Domain Dataset [23]. ENVG00283 contains a transposase DNA-binding domain and is 1,953 bp long. This domain is commonly found at the C-terminus of a large number of transposase proteins. ENVG00294 contains a DNA polymerase III gamma and tau subunit domain and is 1,551 bp long and ENVG00066 contains a methyltransferase type FkbM family domain and is 908 bp long. 

**Table 6:** CDSs with functional predictions identified in both EhV-84 and EhV-86 genomes [1]^†^.

**CDS**	**EhV-86 homologs (putative function/feature)**	**Identity to EhV-86 homologue (%)**
ENVG 00127	ehv014 Longevity-assurance (LAG1) family protein	100
ENVG 00131	ehv018 flap endonuclease-1	100
ENVG 00133	ehv020 putative proliferating cell nuclear antigen	99.61
ENVG 00134	ehv021 putative serine protease	100
ENVG 00135	ehv022 phosphoglycerate mutase family protein	99.07
ENVG 00136	ehv023 putative deoxycytidylate (dCMP) deaminase	98.27
ENVG 00139	ehv026 ribonucleoside-diphosphate reductase small subunit	99.38
ENVG 00142	ehv028 putative lipase	100
ENVG 00144	ehv030 putative DNA polymerase delta catalytic subunit	100
ENVG 00145^1^	ehv031 putative sterol desaturase	100
ENVG 00149	ehv035 putative membrane protein	100
ENVG 00156	ehv041 putative endonuclease	58.33
ENVG 00165^1^	ehv050 serine myristoyl transferase	100
ENVG 00176	ehv060 putative lectin protein	100
ENVG 00177^2^	ehv061 putative fatty acid desaturase	100
ENVG 00178	ehv062 putative membrane protein	100
ENVG 00180	ehv064 DNA-dependent RNA polymerase II largest subunit	100
ENVG 00181	ehv064 DNA-dependent RNA polymerase II largest subunit beta	100
ENVG 00194^1^	ehv077 putative transmembrane fatty acid elongation protein	100
ENVG 00196^1^	ehv079 putative lipid phosphate phosphatase	100
ENVG 00202	ehv085 major capsid protein	99.81
ENVG 00205	ehv088 putative membrane protein	99.02
ENVG 00382	ehv101 putative hydrolase	100
ENVG 00380	ehv103 putative vesicle-associated membrane protein	100
ENVG 00379	ehv104 putative DNA helicase	99.81
ENVG 00378	ehv105 transcription factor S-II (TFIIS) family protein	100
ENVG 00375	ehv108 putative DNA-directed RNA polymerase subunit	100
ENVG 00374	ehv109 OTU-like cysteine protease	100
ENVG 00373	ehv110 putative RING finger protein	100
ENVG 00370	ehv113 bifunctional dihydrofolate reductase-thymidylate synthase	99.79
ENVG 00367	ehv116 putative membrane protein	100
ENVG 00366	ehv117 putative phosphate permease/ sodium-phosphate symporter	100
ENVG 00356	ehv128 ERV1/ALR family protein	98.22
ENVG 00353	ehv131 putative membrane protein	95.08
ENVG 00351	ehv133 putative ATP-dependent protease proteolytic subunit	97.90
ENVG 00348	ehv136 putative nucleic acid-binding protein	98.58
ENVG 00293	ehv137 putative membrane protein	24.90
ENVG 00429	ehv151 putative serine protease	96.94
ENVG 00399	ehv166 putative RING finger protein	97.93
ENVG 00400	ehv167 putative DNA-directed RNA polymerase subunit	100
ENVG 00413	ehv179 Major Facilitator Superfamily protein/transporter	99.63
ENVG 00423	ehv187 putative membrane protein	72.00
ENVG 00445	ehv192 putative membrane protein	94.06
ENVG 00478	ehv207 putative membrane protein	100
ENVG 00287	ehv230 putative endonuclease V	99.22
ENVG 00307	ehv246 putative lectin protein	96.46
ENVG 00232	ehv315 putative membrane protein	100
ENVG 00264	ehv349 putative protease	100
ENVG 00273	ehv358 putative thioredoxin	98.73
ENVG 00276	ehv361 putative serine protease	97.14
ENVG 00278	ehv363 putative esterase	97.72
ENVG 00002	ehv364 putative membrane protein	100
ENVG 00295	ehv364 putative membrane protein	34.55
ENVG 00035	ehv397 putative deoxyuridine 5'-triphosphate nucleotidohydrolase	100
ENVG 00037	ehv399 putative DNA-directed RNA polymerase subunit	100
ENVG 00039	ehv401 putative ribonuclease Hll	99.52
ENVG 00054^2^	ehv415 putative delta 9 acyl- lipid fatty acid desaturase	100
ENVG 00055	ehv416 putative membrane protein	100
ENVG 00070	ehv428 putative ribonucleoside-diphosphate reductase protein	98.79
ENVG 00074	ehv431 putative thymidylate kinase	99.69
ENVG 00077	ehv434 putative DNA-directed RNA polymerase II subunit B	99.74
ENVG 00083	ehv440 putative proliferating cell nuclear antigen	100
ENVG 00087	ehv444 putative DNA topoisomerase	99.64
ENVG 00091	ehv447 putative serine protease	100
ENVG 00095	ehv451 putative protein kinase	100
ENVG 00097	ehv453 putative mRNA capping enzyme	99.47
ENVG 00100	ehv455 putative sialidase	100
ENVG 00104	ehv459 putative nucleic acid independent nucleoside triphosphatase	100
ENVG 00111	ehv465 putative thioredoxin protein	100

^†^ including their sequence homologs (coding sequences) in EhV-84 based on TBLASTN (translated nucleotide database)

^1^ genes involved in sphingolipid biosynthesis

^2^ genes encoding desaturases

## Sphingolipid biosynthesis

EhV-84 shares the same sphingolipid LCB biosynthetic machinery as EhV-86 (all predicted components share 100% sequence identity, see Table 6). Interestingly, like EhV-86, EhV-84 also lacks a critical sphingolipid LCB biosynthetic activity, 3-ketosphinganine reductase [19]. There is now increasing evidence to suggest that these viral sphingolipid genes encode proteins that act in conjunction with the algal host sphingolipid biosynthetic genes to generate bioactive lipid(s). Indeed, ehv050 has been shown to encode a functional serine myristoyl transferase, and its expression has been observed under both laboratory and natural environmental conditions [24-26]. The perfect conservation of these genes suggests both a strong selection pressure and/or a relatively recent shared history between these EhV-84 and EhV-86 genes. The presence of the sphingolipid pathway on coccolithovirus genomes emphasizes the important co-evolutionary dynamics that occur within natural oceanic communities: the genes are examples of horizontal gene transfer events between the viruses and their host.

### Phylogeny: DNA pol and MCP

Two genes, encoding DNA polymerase (DNA pol) and the capsid protein (MCP) have been extensively used as marker genes for different EhV strains within the phycodnavirus family and for the study of coccolithovirus diversity [24,27,28]. In EhV-86 the MCP gene (ehv085) is 1,602 bp long and DNA pol (ehv030) is 3,039 bp long. These protein coding sequences are often viewed as the viral kingdom’s equivalent to 16S rDNA marker genes in bacteria, and are therefore commonly used in phylogenetic studies (Figure 1) [29]. DNA pol seems to be highly conserved in coccolithoviruses. For instance, despite their large size, ehv030 in the reference genome of EhV-86 and its homolog ENVG00144 in EhV-84 share a 100% identity to each other at the nucleotide level. In contrast, the MCP gene of EhV-86 (ehv085) and its homolog in EhV-84 (ENVG00202) are more variable, particularly in the 5′ and 3′ regions. Associated structural differences in MCP as a consequence of this variation may form the bases of the phenotypic diversity displayed by the coccolithoviruses with regards to host range. Such structural differences may also benefit the virus in its purpose of successfully infecting and attaching to the targeted host cells. The evolutionary arms race between the host and the virus is something that the virus must take into account and adapt to; and this might explain why this gene is so variable between strains. These two common marker genes reveal an interesting pattern between EhV-86 and EhV-84. On the whole, the genomes are highly similar, yet subtle and some large (and potentially crucial) genetic differences do occur. The apparent difference in evolutionary divergence rates of core components such as DNA pol and MCP genes is intriguing and suggests that lateral transfer of material between different coccolithovirus genomes may be prevalent in the natural environment. The DNA pol gene may have a more recent shared evolutionary history than its MCP counterpart in the EhV-86/EhV-84 system. Through the sequencing of further strains we hope to shed light on this intriguing dynamic.

## Conclusions

EhV-84 is the fourth member of the coccolithovirus family to be sequenced to date. The genome reveals novel putative protein coding sequences, many of which have no current matches in the sequence databases. Many of the CDSs identified display high conservation with their counterparts in EhV-86, while a handful of highly variable CDSs suggest roles in evolutionary adaptation to their hosts and environment. Further sequencing of related strains will no doubt reveal more about the genetic and functional diversity of these cosmopolitan and environmentally important viruses.
